# Hypoxia-inducible factor 1α (HIF-1α) and reactive oxygen species (ROS) mediates radiation-induced invasiveness through the SDF-1α/CXCR4 pathway in non-small cell lung carcinoma cells

**DOI:** 10.18632/oncotarget.3535

**Published:** 2015-03-12

**Authors:** Qing Gu, Yan He, Jianfeng Ji, Yifan Yao, Wenhao Shen, Jialin Luo, Wei Zhu, Han Cao, Yangyang Geng, Jing Xu, Shuyu Zhang, Jianping Cao, Wei-Qun Ding

**Affiliations:** ^1^ School of Radiation Medicine and Protection and Jiangsu Provincial Key Laboratory of Radiation Medicine and Protection, Medical College of Soochow University, Suzhou, China; ^2^ Collaborative Innovation Center of Radiation Medicine of Jiangsu Higher Education Institutions and School for Radiological and Interdisciplinary Sciences (RAD-X), Soochow University, Suzhou, China; ^3^ Department of Radiation Oncology, Zhejiang Cancer Hospital, Hangzhou, China; ^4^ Department of Radiotherapy, Changzhou Tumor Hospital, Soochow University, Changzhou, China; ^5^ Department of Pathology, University of Oklahoma Health Sciences Center, Oklahoma, United States

**Keywords:** non-small cell lung cancer (NSCLC), ionizing radiation, reactive oxygen species (ROS), CXCR4, invasiveness

## Abstract

Radiotherapy is an important procedure for the treatment of inoperable non-small cell lung cancer (NSCLC). However, recent evidence has shown that irradiation can promote the invasion and metastasis of several types of cancer, and the underlying mechanisms are not fully understood. This study aimed to investigate the molecular mechanism by which radiation enhances the invasiveness of NSCLC cells. We found that after irradiation, hypoxia-inducible factor 1α (HIF-1α) was increased and translocated into the nucleus, where it bound to the hypoxia response element (HRE) in the *CXCR4* promoter and promoted the transcription of *CXCR4*. Furthermore, reactive oxygen species (ROS) also plays a role in the radiation-induced expression of CXCR4. Our results revealed that 2 Gy X-ray irradiation promoted the metastasis and invasiveness of H1299, A549 and H460 cells, which were significantly enhanced by SDF-1α treatment. Blocking the SDF-1α/CXCR4 interaction could suppress the radiation-induced invasiveness of NSCLC cells. The PI3K/pAkt and MAPK/pERK1/2 pathways were found to be involved in radiation-induced matrix metalloproteinase (MMP) expression. *In vivo*, irradiation promoted the colonization of H1299 cells in the liver and lung, which was mediated by CXCR4. Altogether, our findings have elucidated the underlying mechanisms of the irradiation-enhanced invasiveness of NSCLC cells.

## INTRODUCTION

Non-small cell lung cancer (NSCLC) remains a major cause of cancer-related deaths worldwide [1]. Although surgical resection is the main treatment for early-stage NSCLC, radiotherapy plays an important role in patients who cannot not tolerate curative surgery because of advanced age or pulmonary or cardiovascular disease [2,3]. However, local recurrence and distant metastasis frequently occur after radiation therapy, which have become the greatest barrier in the management of cancer [3, 4]. The mechanism of local recurrence and distant metastasis remains a complex issue. Multiple factors have been reported to be involved in cancer metastasis, including tumor-stromal interactions and inflammatory and immune reactions [5-8]. The pro-metastatic effect of radiation has been reported for several types of cancer, including thyroid tumors, Lewis lung cancer, prostate small-cell carcinoma and melanoma [9-12]. The interactions between cancer cells and the surrounding stromal fibroblasts modulated by irradiation have been suggested to be involved in tumor invasion and metastasis [13-15]. However, the mechanism that mediates radiation-induced invasiveness remains largely unknown.

Because the proliferation rate of tumor cells is much higher than that of normal cells, the metabolism of tumor cells is aberrantly accelerated and tumor vessel formation is relatively insufficient, eventually leading to a hypoxic microenvironment in solid tumors [16-17]. Hypoxia-inducible factor 1α (HIF-1α), which is induced by hypoxia, confers protection against cell death and plays an adaptive role. After binding to its cognate enhancer sequence in the promoters of its target genes, HIF-1α regulates the transcription of multiple genes in tumor cells and their surrounding stromal cells. Elevated HIF-1α expression is associated with tumor metastasis, resistance to therapy and poor survival [18, 19]. The radioresistance of tumor cells is closely related to the hypoxia status and is considered to be a major obstacle in radiotherapy [20-22].

Stromal cell-derive d factor 1α (SDF-1α), also known as C-X-C motif chemokine 12 (CXCL12), can be secreted by fibroblasts exposed to irradiation or hypoxic conditions [23]. C-X-C chemokine receptor type 4 (CXCR4) is the receptor for SDF-1α. The SDF-1α-CXCR4 interaction promotes tumor progression through the activation of various pathways, including the JAK/STAT, Rho/Rock, PI3K/Akt and MAPK pathways [24]. Inhibition of the interaction between SDF-1α and CXCR4 by AMD3100 can prevent tumor re-growth, invasion and chemoresistance [25]. CXCR4 is overexpressed in NSCLC tissues [26]. CXCR4 from NSCLC cells promotes the metastatic spread to sites that express high levels of SDF-1α, such as the brain, bone marrow and liver [26]. Whether irradiation can modulate CXCR4 expression and the consequent metastasis of NSCLC cells remains unknown. In this study, we investigated CXCR4 expression and the invasive behavior of NSCLC cells in response to ionizing radiation and its underlying mechanisms.

## RESULTS

### Radiation induced the expression of HIF-1α and CXCR4 in H1299 cells

**Figure 1:**
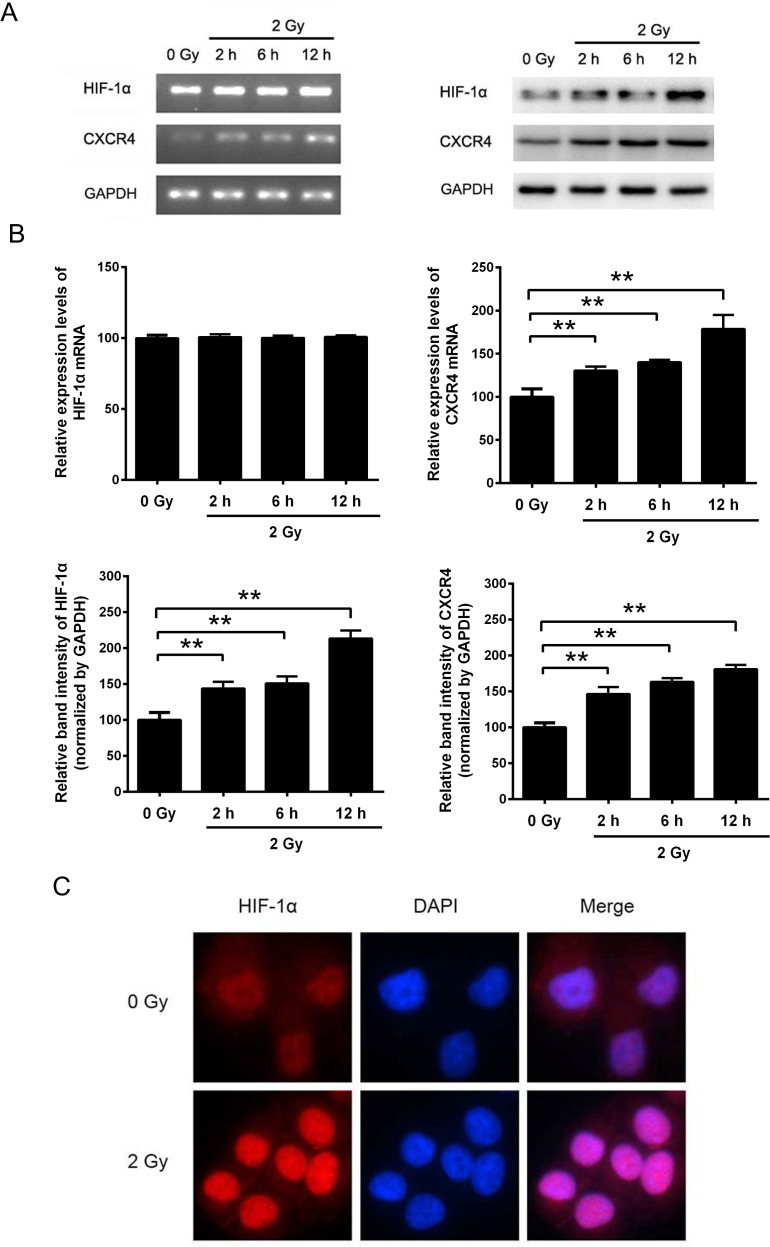
The expression levels of HIF-1α and CXCR4 in H1299 cells were increased by X-ray irradiation (A) H1299 cells were exposed to sham irradiation or 2 Gy X-ray irradiation. Cells were collected 2, 4 and 12 h post-irradiation. The mRNA levels of *HIF-1α* and *CXCR4* were determined by RT-PCR, and the protein expression levels were measured by Western blot analysis. (B) Quantification of HIF-1α and CXCR4 expression with or without irradiation. The quantification results were analyzed using ImageJ image analysis software (NIH, Bethesda, MD). The data are presented as the means ± SEM and normalized to the control cells, * *P* < 0.05; ** *P* < 0.01. (C) Immunofluorescence assay showing the expression and distribution of HIF-1α after irradiation. Cells were immunostained with an anti-HIF-1α and a TRITC-conjugated secondary antibody. Nuclei (blue) were stained with DAPI. All the fluorescence pictures were acquired using the same exposure time.

To explore the effect of irradiation on HIF-1α and CXCR4 expression, NSCLC H1299 cells were exposed to 2 Gy X-ray irradiation. RT-PCR and Western blot analysis revealed that irradiation significantly upregulated CXCR4 expression at both the mRNA and protein levels (Figure 1A and 1B). In contrast, the expression of HIF-1α was significantly upregulated after irradiation at the protein level but not at the mRNA level (Figure 1A and 1B). As shown in Figure 1C, ionizing radiation increased HIF-1α expression and facilitated the translocation of HIF-1α into the cell nucleus, as shown by the increased fluorescence intensity. Altogether, these results suggest that ionizing radiation can increase the HIF-1α protein level and that HIF-1α serves as a nuclear transcription factor and may regulate the expression of CXCR4.

### HIF-1α and ROS were involved in radiation-induced CXCR4 overexpression

**Figure 2:**
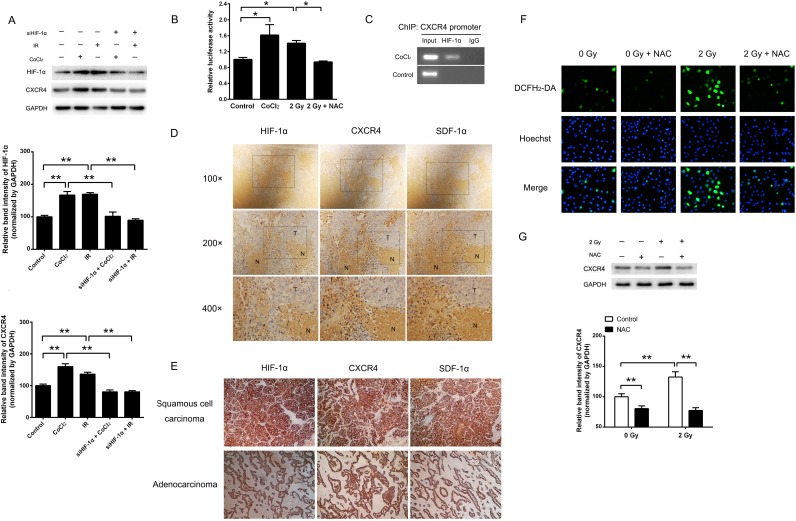
Ionizing radiation enhanced CXCR4 expression through HIF-1α (A) Cells were exposed to the indicated treatments. The expression levels of HIF-1α, CXCR4 and the internal control GAPDH were determined by Western blot analysis. The expression of CXCR4 was upregulated by CoCl_2_- and X-ray irradiation (IR)-induced HIF-1α expression, whereas CXCR4 expression was reduced by HIF-1α knock-down (siHIF-1α). The HIF-1α and CXCR4 expression levels were quantified using ImageJ image analysis software. The data are presented as the means ± SEM and normalized to the control cells, * *P* < 0.05; ** *P* < 0.01. (B) A luciferase reporter containing the *CXCR4* promoter was transfected into H1299 cells, which were then exposed to CoCl_2_, 2 Gy irradiation or 2 Gy irradiation plus NAC. (C) ChIP analysis of HIF-1α binding in H1299 cells. The presence of HIF-1α at the *CXCR4* promoter was verified by PCR. Immunohistochemistry assays were used to detect the expression and co-localization of HIF-1α, SDF-1α and CXCR4 in (D) H1299 xenografts in nude mice and (E) resected tissue sections of NSCLC tumors. (F) Determination of the ROS levels in H1299 cells treated with 2 Gy irradiation or NAC. The fluorescent signals, reflecting the concentration of ROS, were measured using a fluorescence microscope under the same conditions. (G) Radiation increased CXCR4 expression, and treatment with the mTOR inhibitor NAC abolished the CXCR4 protein level induced by irradiation. The CXCR4 expression level was quantified using the ImageJ software. The data are presented as the means ± SEM and normalized to the control cells, * *P* < 0.05; ** *P* < 0.01.

To investigate whether the expression of CXCR4 is regulated by HIF-1α, H1299 cells were treated with the HIF-1α inducer CoCl_2_ or 2 Gy irradiation. The results demonstrated that the expression of CXCR4 was significantly increased after CoCl_2_ treatment or exposure to 2 Gy irradiation (Figure 2A). The luciferase assay confirmed that either CoCl_2_ or 2 Gy irradiation could also increase the luciferase activity of the *CXCR4* promoter containing the reporter (Figure 2B), indicating transcriptional activation of CXCR4. When pre-transfected with a siRNA that targets HIF-1α (siHIF-1α), the hypoxia or radiation-induced CXCR4 expression was abolished (Figure 2A). As shown in Figure 2C, the direct binding of HIF-1α to the *CXCR4* promoter in cells exposed to hypoxia was confirmed by a ChIP assay, suggesting that the CXCR4 expression was modulated by HIF-1α.

We next investigated whether HIF-1α, CXCR4 and SDF-1α are co-expressed *in vivo*. H1299 xenografts in nude mice and lung cancer tissues from patients were used for immunohistochemistry to detect the expression and distribution of HIF-1α, CXCR4 and SDF-1α. As shown in Figure 2D, an intense positive signal was observed in the tumor tissue (T) of the xenografts, whereas weak signals were detected in necrotic areas (N). Significantly higher expression levels of HIF-1α, CXCR4 and SDF-1α were observed in the peripheral areas of necrotic tissues, which were considered to be the hypoxic regions of the solid tumors. A similar expression pattern was also observed in 10 Gy X-ray irradiated H1299 xenografts (data not shown). High expression levels of HIF-1α, CXCR4 and SDF-1α were also detected in both lung adenocarcinoma and squamous cell carcinoma (Figure 2E). Altogether, these results strongly indicate that the expression patterns of HIF-1α, CXCR4 and SDF-1α are related *in vivo*.

Because radiation can generate a variety of ROS, we next explored whether radiation-induced CXCR4 expression is mediated by ROS. As shown in Figure 2F and 2G, after 2 Gy X-ray exposure, the intracellular ROS level was increased, accompanied by elevated CXCR4 expression. However, treatment of NAC, an established ROS scavenger, could neutralize CXCR4 induction. Furthermore, additional of NAC also reduced the transcriptional activation of *CXCR4* promoter by 2 Gy irradiation (Figure 2B). Because NAC is also reported to be an inhibitor of the mammalian targets of the rapamycin (mTOR) [28], which can induce the expression of HIF-1α, we investigated whether radiation-induced CXCR4 expression is mediated by mTOR. As shown in Supplementary Figure 1A, treatment with NAC, rapamycin or NAC plus rapamycin inhibited the phosphorylation of mTOR. However, rapamycin treatment showed no efect on the expression of HIF-1α or CXCR4 after irradiation (Supplementary Figure 1B), suggesting that mTOR is not involved in radiation-induced HIF-1α and CXCR4 expression. The above results indicated that when H1299 cells are exposed to irradiation, ROS may act as an inducing molecule, stimulating CXCR4 expression.

### The impact of the SDF-1α/CXCR4 pathway on cell viability

**Figure 3:**
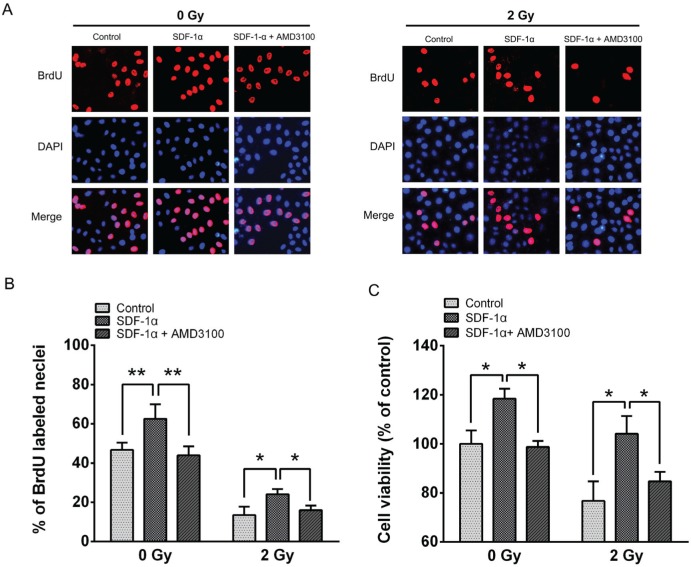
The impact of the SDF-1α/CXCR4 pathway on H1299 cell proliferation (A) Representative image of BrdU-positive cells from a BrdU incorporation assay as described in Materials and Methods. The images were acquired using a fluorescence microscope under the same conditions. (B) The percentage of BrdU-positive cells in the indicated groups. (C) The viability of H1299 cells was assessed using the MTT assay after treatment with SDF-1α and AMD3100 with or without irradiation. The data are presented as the means ± SEM and normalized to the control cells, * *P* < 0.05; ** *P* < 0.01.

To further evaluate the consequences of radiation-induced CXCR4 expression, we conducted a BrdU incorporation assay and an MTT assay to evaluate the changes in cell proliferation. The results revealed that 46.7 ± 3.67% of the H1299 cells in the control group were BrdU positive, whereas 62.6 ± 7.35% of the cells were BrdU positive in the 200 ng/mL SDF-1α-treated group (Figure 3A and 3B). After 2 Gy X-ray irradiation, the percentage of BrdU-positive cells decreased to 13.47 ± 4.31%, and treatment with SDF-1α increased the percentage of BrdU-positive cells to 24.10 ± 2.66%. AMD3100, a specific inhibitor of SDF-1α/CXCR4, significantly blocked BrdU incorporation in both non-irradiated and 2 Gy X-ray-irradiated cells. The MTT assay demonstrated that 96 h after 2 Gy X-ray irradiation, cell viability was reduced. SDF-1α noticeably increased the cell viability in both the sham-irradiated and 2 Gy X-ray-irradiated cells (Figure 3C), which is consistent with the results from the BrdU assay. Moreover, AMD3100 significantly suppressed the proliferation-promoting effect of SDF-1α in both the non-irradiated and irradiated cells. The above results suggest that the H1299 cells treated with SDF-1α exhibited enhanced DNA replication and increased proliferation under both non-irradiated and 2 Gy X-ray-irradiated conditions. Blocking the interaction between SDF-1α and its receptor CXCR4 with AMD3100 abolished the stimulatory effect on proliferation in H1299 cells.

### Ionizing radiation increased the invasiveness of NSCLC cells via the SDF-1α/CXCR4 pathway

**Figure 4:**
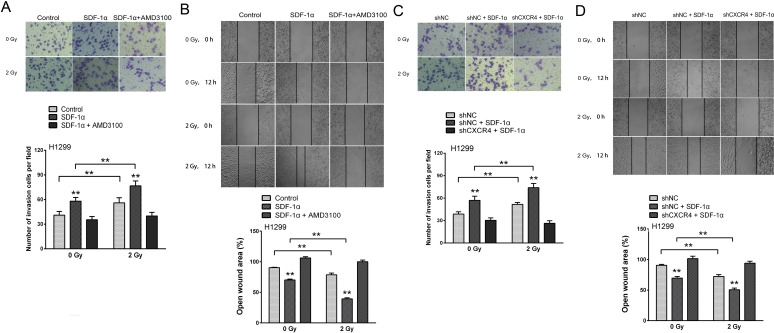
Ionizing radiation increased the invasiveness of H1299 cells via the SDF-1α/CXCR4 pathway (A) Matrigel invasion assay of H1299 cells treated with irradiation, SDF-1α or a combination of irradiation plus SDF-1α. After the treatment for 12 h, the H1299 cells that migrated to the bottom surface of the membrane were stained with Giemsa, and the number of migrated cells was calculated manually. (B) Wound healing assay of H1299 cells treated with irradiation, SDF-1α or a combination of irradiation plus SDF-1α. Wound healing was observed 12 h after the treatment, and the open wound area was normalized to the area at the initial time that the wound was made. The data are presented as the means ± SEM and normalized to the control cells. (C) Matrigel invasion assay of H1299 cells after transfection with an empty vector or CXCR4 shRNA (shCXCR4) followed by treatment with SDF-1α or irradiation. After incubation for 12 h, H1299 cells that migrated to the bottom surface of the membrane were stained with Giemsa, and the migrated cell number was calculated manually. (D) Wound healing assay of H1299 cells after transfection with an empty vector or CXCR4 shRNA (shCXCR4) followed by treatment with SDF-1α or irradiation. Wound healing was observed 12 h after the treatment, and the open wound area was normalized to the area at the initial time that the wound was made. The data are presented as the means ± SEM and normalized to the control cells. * *P* < 0.05; ** *P* < 0.01.

Cancer metastasis is a complex process that involves cell migration and invasiveness. Matrigel invasion assays were performed to explore the effect of ionizing radiation on the invasiveness of H1299 cells. After treatment for 12 h, the H1299 cells that migrated to the bottom surface of the membrane were stained with Giemsa and the number of invading cells was calculated manually. As shown in Figure 4A, irradiation or SDF-1α treatment increased the ability of H1299 cells to invade through the Matrigel and membrane compared with the control cells. Additionally, when treated with SDF-1α and irradiation, the H1299 cells demonstrated a significant 1.87-fold increase in the number of invading cells compared with the control cells, indicating the highest invasive potential (Figure 4A). SDF-1α- and/or irradiation-induced invasiveness was abrogated by AMD3100, indicating the involvement of the SDF-1α/CXCR4 interaction (Figure 4A). To confirm this involvement, CXCR4 knock-down by a shRNA also attenuated SDF-1α- and irradiation-induced cell invasion (Figure 4C).

Because increased motility is also an important characteristic of metastatic cells [6, 8], H1299 cells were subjected to an *in vitro* wound healing assay. Confluent H1299 cell cultures were scraped to create a wound, and cell migration was assessed 12 h later. As shown in Figure 4B, the addition of SDF-1α noticeably reduced the wound area. 2 Gy X-ray irradiation alone also significantly promoted cell migration and the cells treated with both SDF-1α and irradiation demonstrated the narrowest wound area (43.70 % of the control group), suggesting that irradiation plays a role in enhancing the migration capability. Consistent with the results of the transwell Matrigel invasion assay, the SDF-1α/CXCR4 specific inhibitor AMD3100 and CXCR4 knock-down significantly suppressed the cell migration rates induced by SDF-1α and irradiation (Figure 4B and 4D). All of these results suggest that SDF-1α/CXCR4 plays an important role in the radiation-induced invasiveness of H1299 cells.

Because SDF-1α stimulates cell growth, we next investigated whether SDF-1α-induced cell migration and invasion is attributed to cell proliferation. H1299 cells were pretreated with the proliferation inhibitor Epothilone B. The results showed that the Epothilone B treatment did not affect the increase in cell migration and invasion resulting from SDF-1α alone or SDF-1α plus irradiation (Supplementary Figure 2A and 2B). These results rule out the possibility that SDF-1α promotes the cell metastatic potential by increasing cell proliferation.

**Figure 5:**
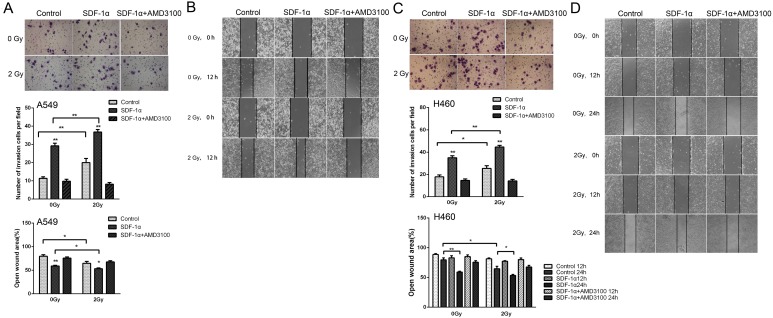
Ionizing radiation increased the invasiveness of A549 and H460 cells Matrigel invasion assay of (A) A549 and (C) H460 cells treated with irradiation, SDF-1α or a combination of irradiation plus SDF-1α. After the treatment for 12 h, the A549 cells that migrated to the bottom surface of the membrane were stained with Giemsa, and the number of migrated cells was calculated manually. Wound healing assay of (B) A549 and (D) H460 cells treated with irradiation, SDF-1α or a combination of irradiation plus SDF-1α. Wound healing was observed 12 or 24 h after the treatment, and the open wound area was normalized to the area at the initial time that the wound was made. The data are presented as the means ± SEM and normalized to the control cells. * *P* < 0.05; ** *P* < 0.01.

To investigate whether radiation-increased invasiveness is specific for H1299 cells, NSCLC A549 and H460 cells was used in wound healing and Matrigel invasion assays. The statistical analysis revealed that 2 Gy X-ray irradiation promoted the invasion and migration of A549 cells 12 h post irradiation (Figure 5A and 5B). Moreover, treatment with AMD3100 inhibited the radiation-induced increase in A549 cell invasion and migration (Figure 5A and 5B). For H460 cells, after incubation for 12 h, we found that the number of cells that invaded through the Matrigel and membrane was increased after 2 Gy irradiation (Figure 5C). In wound healing assay, significantly increased cell migration was found 24 h post irradiation (Figure 5D). These results indicated that ionizing radiation enhanced the invasiveness of NSCLC cells via the SDF-1α/CXCR4 pathway.

### SDF-1α/CXCR4 enhanced the invasiveness of H1299 cells by activating the PI3K/pAkt and MAPK/pERK1/2 pathways

**Figure 6:**
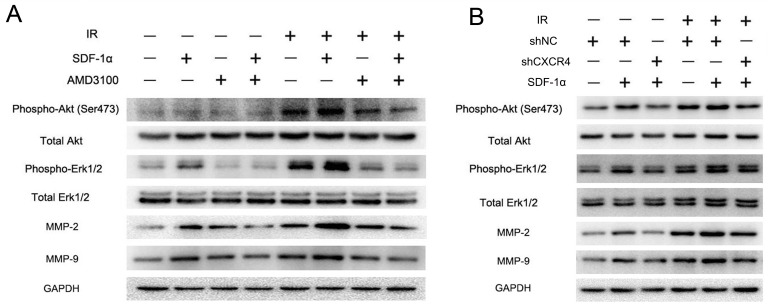
CXCR4 knock-down confirmed that the SDF-1α/CXCR4 interaction played a critical role in the radiation-induced invasiveness of H1299 cells (A) Western blot analysis of the expression levels of downstream molecules in the SDF-1α/CXCR4 pathway, including pAkt, pERK1/2, MMP-2 and MMP-9, after treatment with irradiation, SDF-1α or a combination of SDF-1α plus irradiation. (B) Western blot analysis of the expression of pAkt, pERK1/2, MMP-2 and MMP-9 after the indicated treatment.

The SDF-1α/CXCR4 interaction has been reported to activate several downstream signaling pathways, including the phosphatidylinositol 3-kinase (PI3K)/protein kinase B (Akt) and mitogen-activated protein kinase (MAPK)/ERK1/2 pathways, which then regulate MMP expression [25]. Western blot analysis was performed to investigate the signal transduction involved in the radiation-induced invasiveness of H1299 cells. The results revealed that the CXCR4 ligand SDF-1α could activate the Akt and ERK1/2 pathways by promoting their phosphorylation in H1299 cells (Figure 6A and 6B). The levels of phospho-Akt and phospho-ERK1/2 were also upregulated by 2 Gy irradiation and were further elevated in the cells treated with both SDF-1α and irradiation. As shown in Figure 6A and 5B, SDF-1α- and irradiation-induced phospho-Akt and phospho-ERK1/2 levels were greatly reduced by treatment with the SDF-1α/CXCR4-specific inhibitor AMD3100 or by CXCR4 knock-down (shCXCR4), indicating that the SDF-1α/CXCR4 interaction is required for the radiation-induced effect. Additionally, the phosphorylation of Akt and ERK1/2 increased the expression of MMP-2 and MMP-9, which have been extensively studied previously [29].

**Figure 7:**
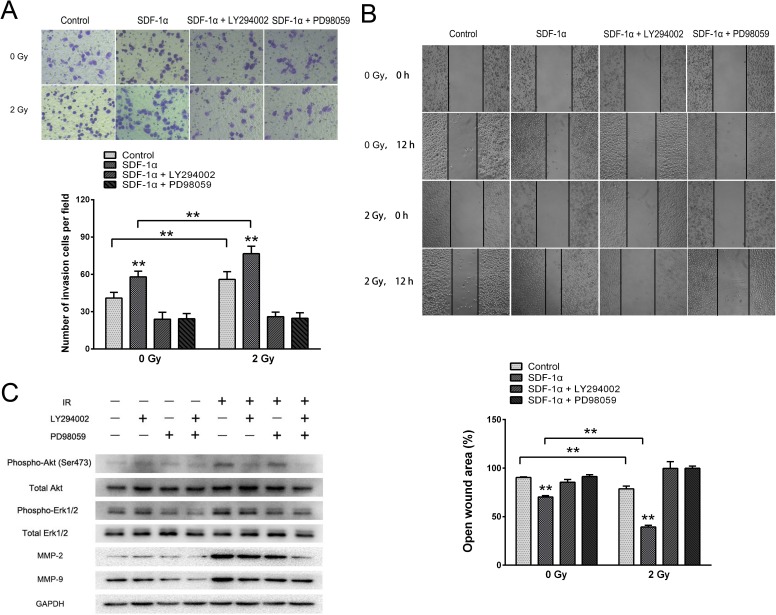
SDF-1α/CXCR4 activated the PI3K/pAkt and MAPK/pERK1/2 pathways, which enhanced the invasiveness of H1299 cells by upregulating MMP expression (A) Matrigel invasion assay of H1299 cells pretreated with LY294002 or PD98059. After the treatment for 12 h, H1299 cells that migrated to the bottom surface of the membrane were stained with Giemsa, and the migrated cell number was calculated manually. (B) Wound healing assay of H1299 cells pretreated with LY294002 or PD98059. Wound healing was observed 12 h after the treatment, and the open wound area was normalized to the area at the initial time that the wound was made. The data are presented as the means ± SEM and normalized to the control cells. * *P* < 0.05; ** *P* < 0.01. (C) Western blot analysis of the expression of pAkt, pERK1/2, MMP-2 and MMP-9 after the treatment.

To further illustrate the importance of these pathways in SDF-1α- and irradiation-induced migration and invasion, the Akt-specific inhibitor LY294002 and the ERK1/2-specific inhibitor PD98059 were used. The Matrigel invasion assay and wound healing migration assay demonstrated that pretreatment with LY294002 or PD98059 abolished the baseline and radiation-induced invasiveness of H1299 cells (Figure 7A and 7B). Western blot analysis showed that the radiation-induced production of MMP-2 and MMP-9 was substantially downregulated by the inhibition of both the Akt and ERK1/2 pathways (Figure 7C). Altogether, these results demonstrate that ionizing radiation-induced invasiveness is mediated by Akt and ERK1/2 phosphorylation and enhanced MMP expression.

### Ionizing radiation increased the metastatic potential of H1299 cells *in vivo*

**Figure 8:**
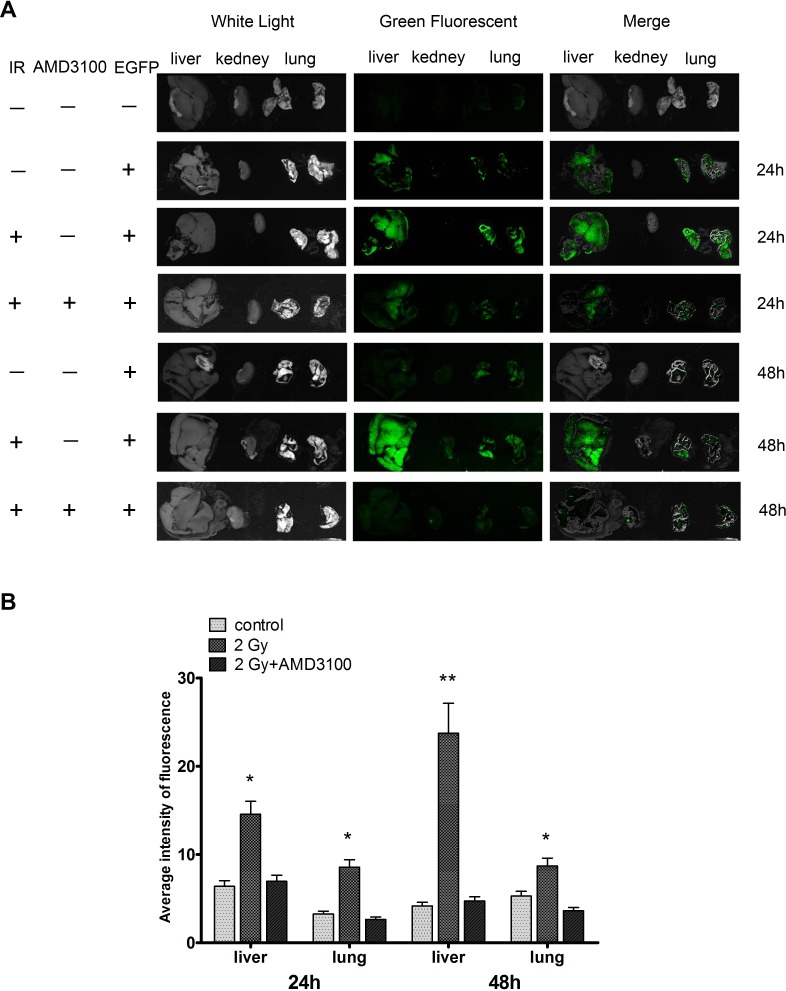
Ionizing radiation increased the colonization of H1299 cells *in vivo* H1299 cells were infected with Ad-EGFP and treated with sham irradiation or 2 Gy X-ray irradiation. Cells were then injected into nude mice via the lateral vein. (A) After 24 h, H1299 cell colonization in the nude mouse organs was visualized using an *in vivo* imaging system. All the photos were taken under the same condition. (B) After cell injection, nude mice were administered AMD3100. Relative green fluorescence levels in the indicated group of tissues were calculated using the ImageJ image analysis software.

We next investigated the consequence of 2 Gy irradiation on the metastatic potential of lung cancer H1299 cells *in vivo*. The tail vein injection of human tumor cells into nude mice is a well-established *in vivo* model for studying tumor metastasis [30]. H1299 cells were infected with Ad-EGFP and treated with sham irradiation or 2 Gy X-ray irradiation. The cells were then injected into nude mice via the lateral tail vein. Approximately 24 h after injection, H1299 cell colonization in the mouse organs was visualized using an *in vivo* imaging system. As shown in Figure 8A, the irradiated cells exhibited a stronger green fluorescence in the livers and lungs of nude mice compared with the sham-irradiated H1299 cells. These results suggest that the irradiation promoted the metastatic potential of the human NSCLC cells. Moreover, inhibition of the CXCR4 pathway by AMD3100 significantly decreased the colonization of the fluorescent cells in these organs (Figure 8B), indicating that radiation-induced cancer cell colonization is mediated by SDF-1α/CXCR4.

## DISCUSSION

**Figure 9:**
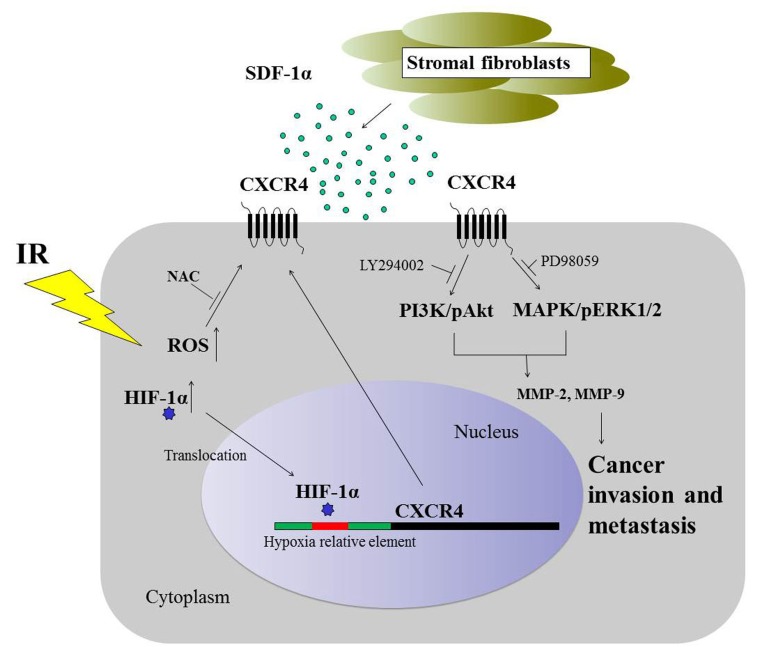
Schematic representation of the signaling pathways involved in the radiation-induced invasiveness of H1299 cells The expression of CXCR4 is regulated by HIF-1α and ROS. Extracellular SDF-1α activates radiation-induced CXCR4, leading to the activation of pAkt and pERK1/2 and an increase in MMP-2 and MMP-9 protein expression, consequently resulting in cancer invasiveness.

Recently, ionizing radiation has been reported to promote the metastasis of several types of cancer cells, but the molecular mechanisms are poorly understood [31-33]. Patel et al. found that radiation enhanced esophageal epithelial cell migration and invasion through a paracrine mechanism that involves stromal-derived hepatocyte growth factor [34]. Another study showed that the K-Ras pathway may be involved in the irradiation-induced cell migration and metastasis of cervical cancer cells [35]. The epithelial-mesenchymal transition (EMT) mediated by TGF-β also plays a critical role in the radiation-enhanced migratory and invasive capabilities of cancer cells [36]. In this study, we found that 2 Gy X-ray irradiation stimulated the invasiveness of H1299 cells upon treatment with SDF-1α. Conversely, the SDF-1α/CXCR4 axis-specific inhibitor AMD3100 and CXCR4 knock-down attenuated the pro-invasive effect of radiation and SDF-1α, indicating that SDF-1α/CXCR4 is critical for the radiation-mediated dissemination of NSCLC cells (Figure 9). The *in vivo* study confirmed that CXCR4 mediates the radiation-induced colonization potential. Our study has elucidated a novel mechanism that drives the radiation-induced invasiveness of cancer cells.

Radiation-induced HIF-1α expression has been shown to be highly relevant to the malignancy of cancer cells. Burrows et al. found that radiation can induce migration through a HIF-dependent manner in FTC133 thyroid carcinoma cells, which can be successfully inhibited by targeting PI3K using GDC-0941 *in vitro* and *in vivo* [9]. Another investigation revealed that the radiation-increased metastatic dissemination of human melanoma xenografts was mediated by the hypoxia-induced upregulation of the urokinase-type plasminogen activator receptor (uPAR) [37]. The induced expression of HIF-1α and its target genes plays a critical role in cell growth, metastasis and resistance to chemotherapy and radiotherapy [38]. In this study, our findings demonstrated that radiation increased HIF-1α expression at the protein level, and radiation-induced HIF-1α expression further upregulated CXCR4 expression. Several studies have reported that both the *SDF-1α* and *CXCR4* promoters contain hypoxia response elements (HREs) and that their expression is upregulated by HIF-1α [25,39-42]. In this study, we found that the expressions of HIF-1α, CXCR4 and SDF-1α were co-localized in the peripheral region of necrotic tumor tissues, which were relatively hypoxic due to reduced blood flow. These results suggest a potential association between CXCR4 and SDF-1α with tumor hypoxia in NSCLC. A functional study in which HIF-1α was knocked down and the ChIP assay confirmed that HIF-1α regulates CXCR4 expression. We further demonstrated that mTOR was also involved in elevating CXCR4 expression.

Previous studies have demonstrated that the SDF-1α/CXCR4 interaction modulates cell migration, invasion and MMP secretion through various downstream signaling pathways, including PI3K/pAkt, MAPK/pERK1/2, JAK/STAT, Rho/Rock, c-Fos/c-Jun, AP-1 and K-Ras [25]. Here, we found that SDF-1α and radiation-induced Akt and ERK1/2 phosphorylation played a key role in the metastasis of NSCLC cells. SDF-1α treatment resulted in a significant increase in Akt and ERK1/2 phosphorylation, and the expression of MMP-2 and MMP-9 was also upregulated. AMD3100 treatment and CXCR4 knock-down resulted in a noticeable inhibition in pAkt, pERK1/2, MMP-2 and MMP-9 expression. The above findings indicate that SDF-1α/CXCR4 could activate the PI3K and MAPK signaling pathways and promote the secretion of matrix metalloproteinases to facilitate the invasion and metastasis of H1299 cells. The activation of Akt and ERK1/2 is known to be involved in cancer cell proliferation, apoptosis, angiogenesis, invasion and metastasis [43,44]. Several studies using different cell types have indicated that Akt and ERK1/2 appear to play a central role in regulating the expression of MMPs [29,45,46]. Inhibition of these pathways has been shown to have potential in preventing angiogenesis and the invasion and metastasis of a wide range of tumors. To investigate the possible mechanisms involved in the metastatic progression of H1299 cells, we examined the MMP-2 and MMP-9 protein levels after treatment with PI3K/pAkt and MAPK/pERK1/2 inhibitors (LY294002 and PD98059), and the reduced expression of the MMPs confirmed the indispensable role of pAkt and pERK1/2 in radiation-induced tumor invasion and metastasis. The results of the present study indicated that pAkt and pERK1/2 mediated the radiation-induced invasiveness of H1299 cells through MMP-2 and MMP-9 expression. Whether other pathways are involved in this process warrants further investigation.

Altogether, we demonstrated that radiation enhanced the metastasis and invasiveness of NSCLC H1299 cells though the HIF-1α/CXCR4 pathway. The interaction between the stroma-derived factor SDF-1α and the increased CXCR4 further enhanced the radiation-induced invasiveness of lung cancer cells through pAkt and pERK1/2 signaling transduction pathways (Figure 9). This study contributes to our understanding of the cellular and molecular changes that occur during radiotherapy. Inhibiting the SDF-1α and CXCR4 interaction may be a potential therapeutic approach for treating radiation-induced cancer cell invasion during radiotherapy for malignant tumors.

## MATERIALS AND METHODS

### Reagents

CoCl_2_, AMD3100 and NAC were purchased from Sigma (St. Louis, MO). The pAkt inhibitor LY294002 and the pERK1/2 inhibitor PD98025 were purchased from Cell Signaling Technology (Beverly, MA). The mTOR inhibitor rapamycin and proliferation inhibitor Epothilone B were obtained from Selleck Chemicals (Houston, TX). The anti-HIF-1α and anti-CXCR4 rabbit monoclonal antibodies were purchased from Abcam (Cambridge, MA). The anti-phospho-Akt, anti-Akt, anti-phospho-ERK1/2, anti-ERK1/2, anti-matrix metalloproteinase 2 (MMP-2), anti-MMP-9 and anti-GAPDH monoclonal antibodies were obtained from Cell Signaling Technology (Beverly, MA). Human CXCR4 short hairpin RNA and HIF-1α small-interfering RNA were obtained from Genepharma (Shanghai, China). The transfection reagent was purchased from Tiangen (Beijing, China).

### Cell culture and irradiation

The human non-small cell lung carcinoma cell lines H1299, A549 and H460 were maintained in RPMI 1640 with 10% (v/v) fetal bovine serum (Gibco, Grand Island, NY) and 1% (v/v) antibiotics (100 U/mL penicillin and 100 μg/streptomycin) at 37°C and a 5% CO_2_ atmosphere. The authenticity of this cell line was confirmed by measuring the STR profile and the P53 expression.

The cells were irradiated with a total dose of 2 Gy using an X-ray linear accelerator (RadSource, Suwanee, GA) at a fixed dose rate of 1.15 Gy/min. Sham-irradiated cells were defined as the 0 Gy group.

### Xenografts of nude mice

Xenograft experiments were conducted in 6-7-week-old female nude mice purchased from Shanghai SLAC Laboratory Animal Co., Ltd. (Shanghai, China). H1299 cells were harvested in PBS, and 2.5×10^6^ cells were injected subcutaneously into the legs of nude mice following standard injection procedures. The procedures for the use and care of animals were approved by the Ethical Committee of Soochow University.

### Reverse transcription-polymerase chain reaction (RT-PCR)

Total RNA from each group of H1299 cells was extracted using TRIzol (Invitrogen, Carlsbad, CA) in accordance with the manufacturer's instructions. For reverse transcription, 1.0 μg of RNA per sample was reverse transcribed using an oligo(dT)_12_ primer and Superscript II reverse transcriptase (Invitrogen). The primers for *HIF-1α*, *CXCR4* and the internal control *glyceraldehyde-3-phosphate dehydrogenase* (*GAPDH*) were as follows: *HIF-1α*, forward: 5′-TCACCACAGGACAGTACAGGATGC-3′ and reverse: 5′-CCAGCAAAGTTAAAGCATCAGGTTCC-3′; *CXCR4*, forward: 5′-AGCTGTTGGCTGAAAAGGTGGTCTATG-3′ and reverse: 5′-GCGCTTCTGGTGGCCCTTGGAGTGTG-3′; and *GAPDH*, forward: 5′-AGCCACATCGCTCAGACA-3′ and reverse: 5′-GCCCAATACGACCAAATCC-3′. RT-PCR was conducted using the 2x Taq PCR Master Mix (Tiangen, Beijing, China) according to the manufacturer's instructions. The quantification of each band was performed using ImageJ software (NIH, Bethesda, MD).

### Immunofluorescence (IF)

Cells were grown on chamber slides and sham irradiated or irradiated with 2 Gy X-ray. After 12 h, the cells were fixed with 4.0% paraformaldehyde and permeabilized with 0.5% Triton X-100 (Sigma, St. Louis, MO) at room temperature. The cells were blocked in 5% bovine serum albumin for 1 h. Then, immunofluorescence was performed as previously described [27] using an anti-HIF-1α antibody (Epitomics, Burlingame, CA) for 12 h at 4°C. The cells were then washed with cold PBS and incubated with TRITC-conjugated goat anti-rabbit IgG (Beyotime, Nantong, China) for 45 min at 37°C. The cells were counter-stained with 4′-6-diamidino-2-phenylindole (DAPI, Invitrogen, Carlsbad, CA) to visualize the nuclei and were observed using a fluorescence microscope.

### Human NSCLC tissues

Lung cancer tissues were collected from the Changzhou Tumor Hospital affiliated with the Medical College of Soochow University (Changzhou, China). All of the patients were diagnosed with histologically confirmed NSCLC, and the histological features of the specimens were evaluated by a senior pathologist. Informed consent for sample collection was obtained from all patients. This study was approved by the Ethical Committee of Changzhou Tumor Hospital and Soochow University. The samples were fixed with formalin and embedded in paraffin.

### Immunohistochemical (IHC) analyses

The expression patterns of HIF-1α, CXCR4 and SDF-1α in human tissue samples and H1299 xenografts in nude mice were analyzed using immunohistochemistry. Tissue paraffin sections were deparaffinized and heat-treated with citrate buffer, pH 6.0, for 7 min as an epitope retrieval protocol. Endogenous peroxidase was blocked with 3% hydrogen peroxide for 15 min at room temperature, and non-specific-binding sites were blocked with 4% skim milk powder for 30 min. Sections were then incubated with the primary antibody for 1 h (dilution 1:200) mixed with 2% skim milk powder to reduce nonspecific staining. Biotinylated secondary antibody was then added for 30 min. An avidin–biotin–peroxidase complex (Beyotime, Nantong, China) was added, and the color was developed using 3-3′-diaminobenzidine. Counterstaining was performed with hematoxylin. All steps were performed at room temperature.

### Chromatin immunoprecipitation Assay (ChIP)

Binding of HIF-1α to the *CXCR4* promoter was analyzed by ChIP assay using a ChIP assay kit (Upstate Biotechnology, Lake Placid, NY). H1299 cells were subjected to hypoxia, fixed in 1% formaldehyde and sonicated. The sonicated cell supernatant was diluted in ChIP dilution buffer and precleared for 30 min with a salmon sperm DNA/protein A agarose slurry. The immunoprecipitations were performed at 4°C overnight with a HIF-1α antibody. The antibody/histone complexes were collected with a salmon sperm DNA/protein A agarose slurry. After proteinase K digestion, the DNA was purified. The immunoprecipitated DNA was then subjected to PCR amplification. The sequences of the promoter-specific primers included the *CXCR4* promoter region between −1124 and −1324 bp (relative to the transcription start site) as follows: (forward) 5′-CCTCTCCTCGGGACCATTTC-3′ and (reverse) 5′-CTGGCTGAGGTTTGCAGTG-3′.

### Western blot analysis

After treatment, cells were harvested and lysed in RIPA lysis buffer (50 mM Tris-HCl, pH 7.4, 150 mM NaCl, 1% Triton X-100, and 1% sodium deoxycholate, 0.1% SDS) that contained a protease inhibitor cocktail (Sigma, St. Louis, MO) for 30 min at 4°C. Forty micrograms of protein from each lysate were fractionated by 10% SDS-PAGE and transferred to polyvinylidene difluoride membranes (Millipore, Bedford, MA). After blocking with 5% nonfat milk in PBS-Tween-20 for 1 h at room temperature, the membranes were blotted with the appropriate primary antibody. GAPDH was used as a loading control. After washing four times with TBST, the membranes were incubated with a horseradish peroxidase (HRP)-conjugated anti-rabbit or anti-mouse secondary antibody (Santa Cruz Technology, Santa Cruz, CA) for 2 h. The proteins were visualized using enhanced chemiluminescence (ECL, Beyotime, Nantong, China).

### Reactive oxygen species (ROS) generation assay

The ROS levels of the H1299 cells were measured using the ROS-sensitive dye 2′,7′-dichlorofluorescein diacetate (DCFH2-DA), which is converted by ROS into the highly fluorescent product 2′,7′-dichlorofluorescein (DCF). H1299 cells were incubated with DCFH2-DA (10 μM) diluted in serum-free medium. After incubation, the concentration of ROS, which was represented as the level of DCF fluorescence, was measured using a fluorescence microscope. The nuclei were counterstained with Hoechst 33342. Quantification of the fluorescence intensity was conducted using ImageJ software (NIH, Bethesda, MD).

### Luciferase assays

A luciferase reporter that contained a fragment of the *CXCR4* promoter (−1400 to −1, relative to the transcription start site) was constructed by Shanghai Sangon Biotech Co., Ltd. (Shanghai, China). For the luciferase assay, 1 μg of the reporter vector with the *CXCR4* promoter was cotranfected with 50 ng of pRL-TK (Promega, Madison, WI) to correct the transfection efficiency. The luciferase activity was measured with the Dual-Luciferase Reporter Assay System (Promega). Promoter activities were expressed as the ratio of *Firefly* luciferase to *Renilla* luciferase activities.

### BrdU incorporation assay

The proliferation rate of H1299 cells was determined by the uptake of 5-bromo-2′deoxyuridine-5′monophosphate (BrdU) into DNA. Cells in the logarithmic growth phase were trypsinized and transferred to chamber slides. H1299 cells were treated with SDF-1α, AMD3100 or a combination of SDF-1α and AMD3100 after 0 or 2 Gy irradiation. Twenty-four hours later, the cells were labeled with 10 μM BrdU (Sigma-Aldrich, St. Louis, MO) for 1 h. Then, the cells were fixed and permeabilized with 0.5% Triton X-100 and 0.1% citric acid. The cells were blocked in 5% bovine serum albumin. Then, immunofluorescence was performed as previously described [32] by incubation with an anti-BrdU antibody (Abcam, Cambridge, MA) for 12 h at 4°C. The cells were then washed with cold PBS and incubated with TRITC-conjugated goat anti-rabbit IgG (Beyotime, Nantong, China) for 45 min at 37°C. The cells were counter-stained with DAPI to visualize the nuclei and observed under a fluorescence microscope (Olympus, Tokyo, Japan).

### Cell viability assay

H1299 cells were trypsinized and seeded onto 96-well plates at a density of 2×10^3^ cells/well for culture. Cell proliferation was measured using the 3-(4,5-dimethylthiazol-2-yl)-2,5-diphenyl-2H-tetrazolium bromide (MTT) assay 96 h after irradiation with 2 Gy X-ray. Briefly, 20 μL of MTT solution (5 mg/mL) was added to each well, and the cells incubated for 4 h at 37°C. Then, the medium was aspirated, and 100 μL dimethylsulfoxide (DMSO) was added. The optical density was measured at 492 nm with a microplate reader (Bio-Rad, Hercules, CA). The viability index was calculated as the experimental OD value/the control OD value. Three independent experiments were performed in quadruplicate.

### Wound healing migration and Matrigel invasion assays

Cells were seeded onto 6-well plates and allowed to form a confluent monolayer for 24 h. After treatment, the monolayer was scratched with the tip of a 200 μL pipette and then washed twice with PBS to remove the floating and detached cells. Then, fresh serum-free medium was added, and photos were taken at 0, 12 and 24 h to assess cell migration using a microscope (Olympus, Tokyo, Japan).

The invasive potential of NSCLC cells was assessed using 24-well Matrigel invasion chambers (pore size 8 μm, Costar, New York, NY). Inserts were pre-coated with 40 μL Matrigel (1:4 dilution; BD Biosciences, San Jose, CA). Then, 5×10^4^ cells/mL NSCLC cells in serum-free medium were added to the upper chambers. The lower chambers were filled with medium that contained 10% fetal bovine serum.

After incubation for 12 h, the cells remaining in the upper chambers were scraped off, and the invading cells in the lower chambers were fixed with 3.7% paraformaldehyde. Then, the cells were stained with Giemsa at room temperature and photographed under a microscope.

### *In vivo* imaging of H1299 cell colonization

Four-week-old female outbreed BALB/c mice were purchased from Shanghai SLAC Laboratory Animal Co., Ltd. (Shanghai, China), and kept under specific pathogen-free conditions. H1299 cells were infected with EGFP overexpression adenovirus (Ad-EGFP). Twenty-four hours after infection, the cells were treated with sham irradiation or 2 Gy X-ray irradiation. Nude mice were intravenously (tail vein) injected with 2 × 10^6^ cells. AMD3100 (5 mg/kg/day) was administrated by intraperitoneal injection, and 24 h later, H1299 cell colonization in the nude mouse organs was visualized using the Kodak *in vivo* imaging system. All the photos were taken under the same condition. The relative fluorescence intensity in the tissues was calculated using the ImageJ image analysis software (MD, USA). The design and implementation of the study were approved by the Ethics Committee, Soochow University.

### Statistical analysis

Data are expressed as the mean ± standard error of the mean (SEM) of at least three independent experiments. The data were analyzed by one-way analysis of variance (ANOVA). Statistical analyses were performed using PRISM version 6.0 (Graph-Pad Software, San Diego, CA). Statistical significance was considered to be a *P*-value < 0.05. The relative mRNA and protein expression levels were analyzed using the ImageJ image analysis software (NIH, Bethesda, MD).

## SUPPLEMENTARY MATERIAL, FIGURES


