# Supplementary material for: Hypoxia-inducible factor 1α (HIF-1α) and reactive oxygen species (ROS) mediates radiation-induced invasiveness through the SDF-1α/CXCR4 pathway in non-small cell lung carcinoma cells
Source: Oncotarget. 2015 Mar 12;6(13):10893–907. doi: 10.18632/oncotarget.3535 (PMC4484427; doi:10.18632/oncotarget.3535)
Supplement: Supplementary file 1 [file oncotarget-06-10893-s001.pdf]

# Hypoxia-inducible factor 1α (HIF-1α) and reactive oxygen species (ROS) mediates radiation-induced invasiveness through the SDF-1α/CXCR4 pathway in non-small cell lung carcinoma cells

**Supplementary Material**

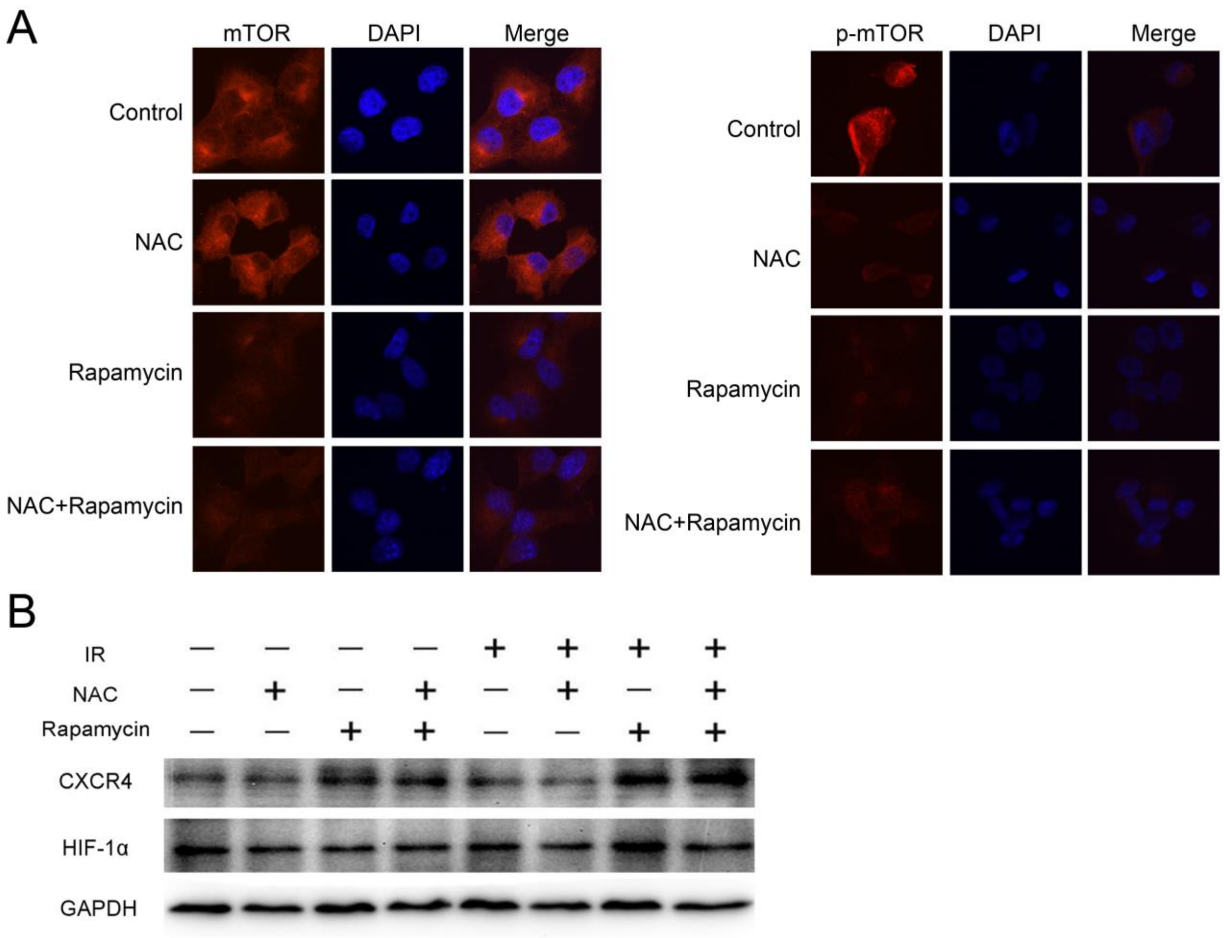


**Supplementary Figure 1: mTOR is not involved in the radiation-induced HIF-1α and CXCR4 expression.** (A) Immunofluorescence assay showing the expression and distribution mTOR and phosphorylated mTOR after treatment of NAC or/and rapamycin. (B) Western blot analysis of HIF-1α and CXCR4 after treatment with NAC or /and rapamycin with or wihout irradiation.

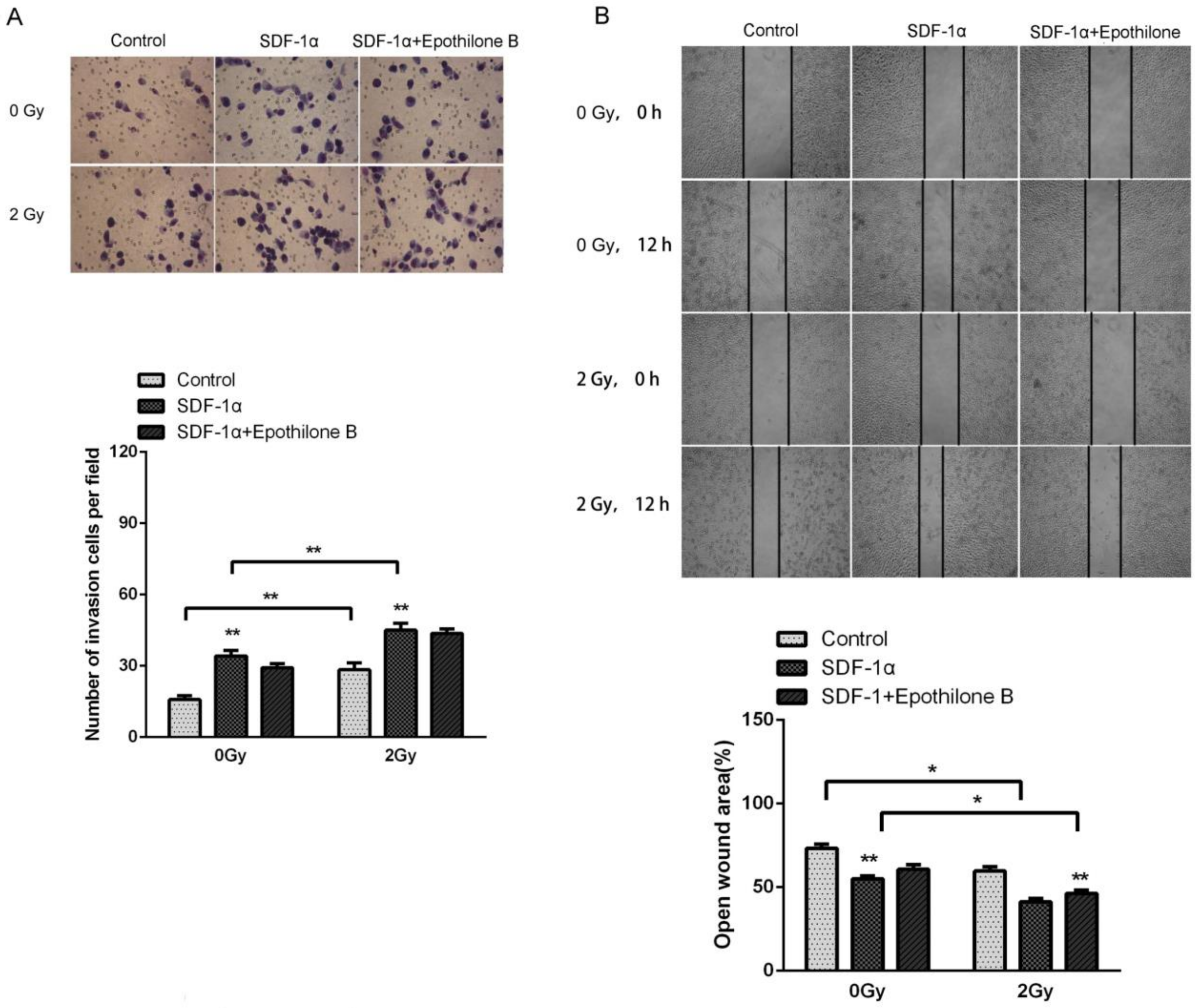


**Supplementary Figure 2: The effect of Epothilone B on cell migration and invasion.** (A) Matrigel invasion assay of H1299 cells treated with SDF-1α or SDF-1α plus Epothilone B prior to 0 or 2 Gy irradiation. After the treatment for 12 h, the H1299 cells that migrated to the bottom surface of the membrane were stained with Giemsa, and the number of migrated cells was calculated manually. (B) Wound healing assay of H1299 cells treated with SDF-1α or SDF-1α plus Epothilone B prior to 0 or 2 Gy irradiation. Wound healing was observed 12 h after the treatment, and the open wound area was normalized to the area at the initial time that the wound was made. The data are presented as the means ± SEM and normalized to the control cells. * $P < 0.05$; ** $P < 0.01$.
